# Supplementary material for: Genetic dissection of regulation by a repressing and novel activating corrinoid riboswitch enables engineering of synthetic riboswitches
Source: mBio. 2023 Oct 12;14(5):e01588-23. doi: 10.1128/mbio.01588-23 (PMC10653944; doi:10.1128/mbio.01588-23)
Supplement: Supplemental figures — Figure S1 through S7 and caption for Table S1. [file mbio.01588-23-s0001.docx]

**Table S1.** Strain list and riboswitch sequences used in this study. Riboswitch DNA sequences include the P1 stem thru the poly-uracil tract. Benchling links are to annotated sequence files for plasmids or ordered eBlocks (IDT).

**
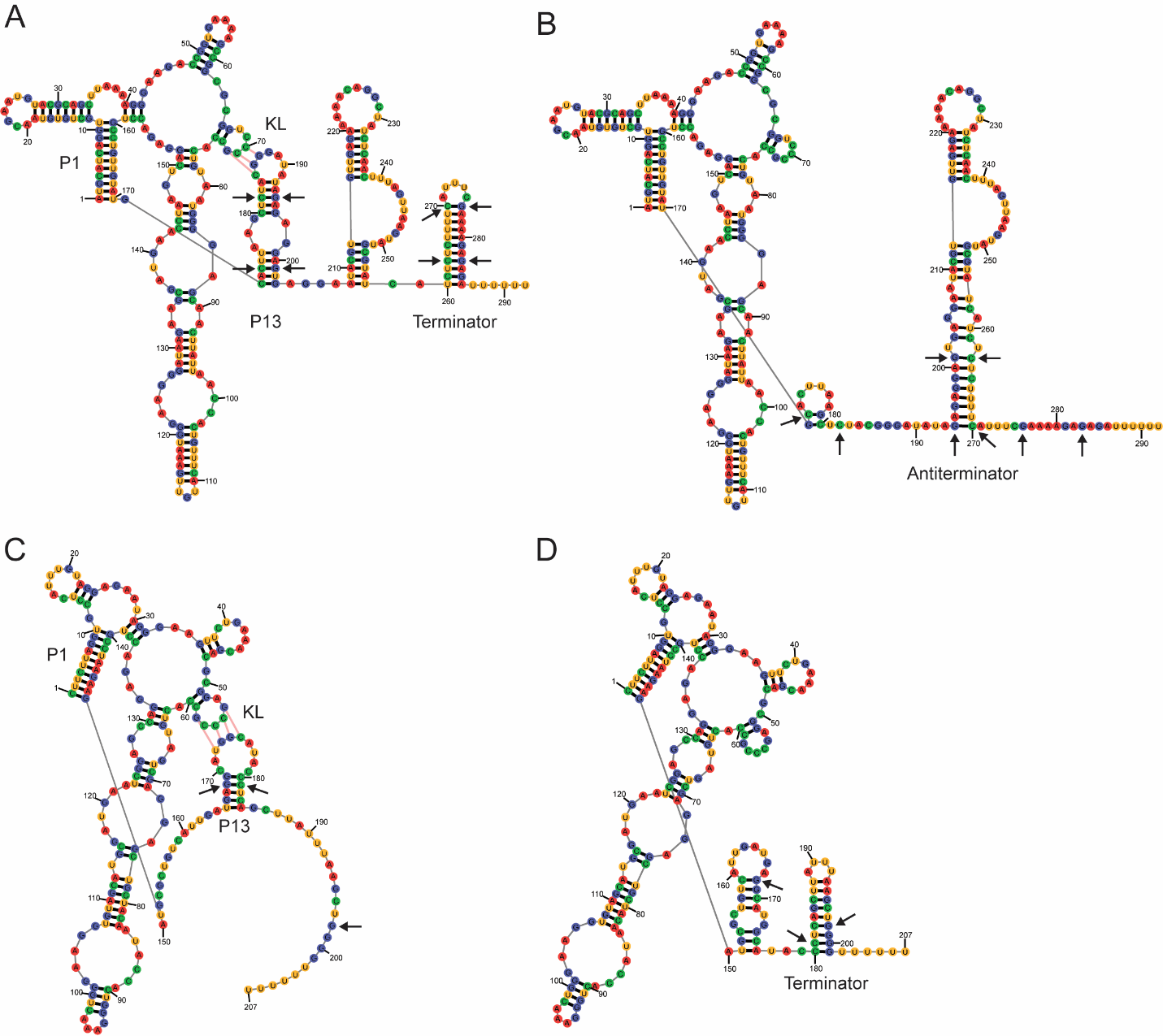
**

**Figure S1.** Sequences and predicted structures of the *P. megaterium metE* and *A. halodurans cobT* riboswitches. Models were constructed manually and guided by computer-generated predictions using the StructureEditor program (22). Bases are color coded (adenine, red; guanine, blue; uracil, yellow; cytosine, green). Black arrows show the locations of the mutations made in this study. (A) The effector-bound state of the *P. megaterium metE* riboswitch. (B) The effector-unbound state of the *P. megaterium metE* riboswitch. (C) The effector-bound state of the *A. halodurans cobT* riboswitch. (D) The effector-unbound state of the *A. halodurans cobT* riboswitch. The KL is represented with pink lines.

**
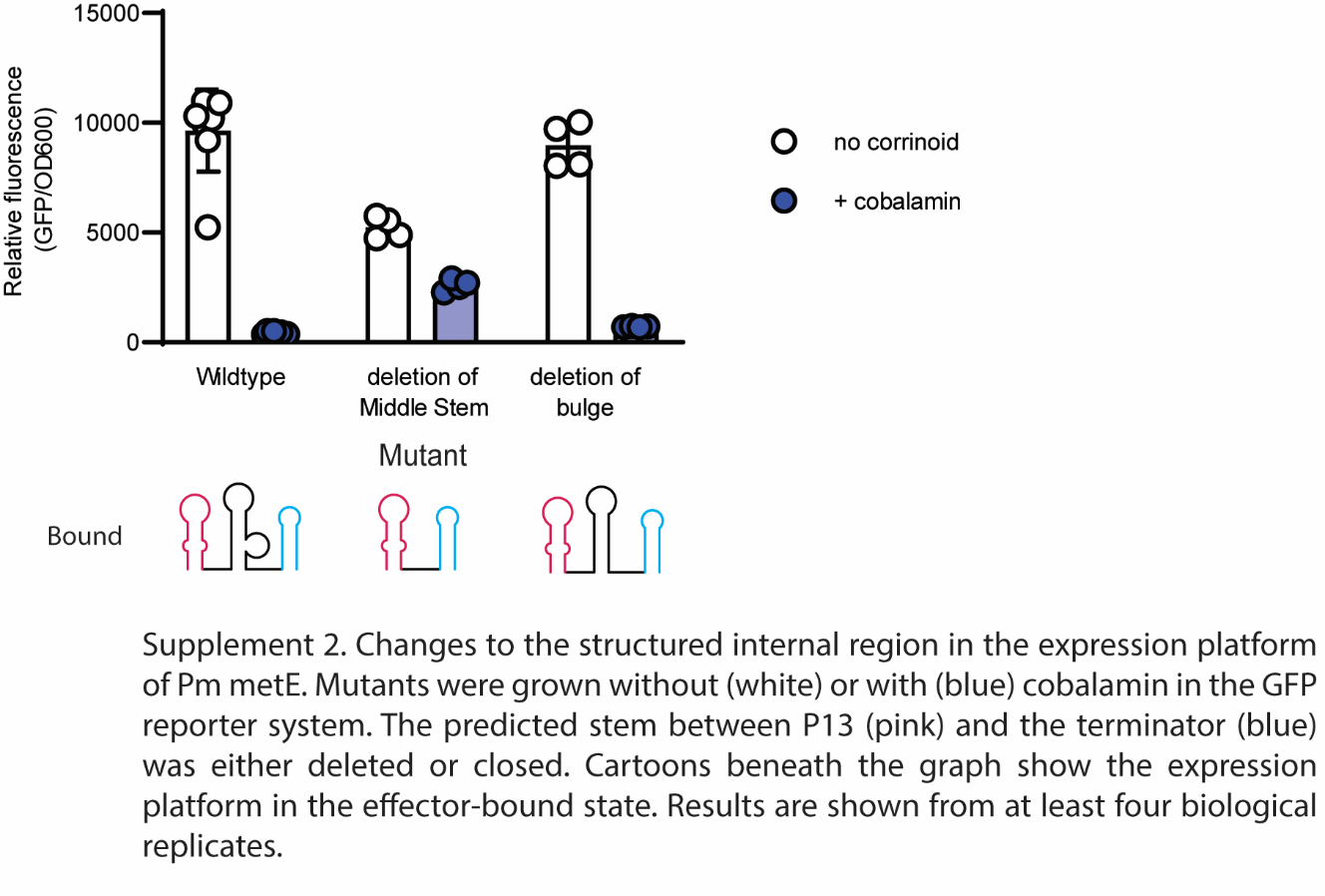
**

**Figure S2.** Changes to the structured internal region in the expression platform of the *P. megaterium metE* riboswitch*.* Gene expression was measured without (white) or with (blue) addition of 100 nM cobalamin in the *B. subtilis* GFP reporter system. The predicted “middle stem” (black) between P13 (pink) and the terminator (blue) was either deleted or closed. Diagrams below the graph show the expression platform in the effector-bound state. Data from four or more biological replicates are shown; bars and error bars represent mean and standard deviation, respectively.

**
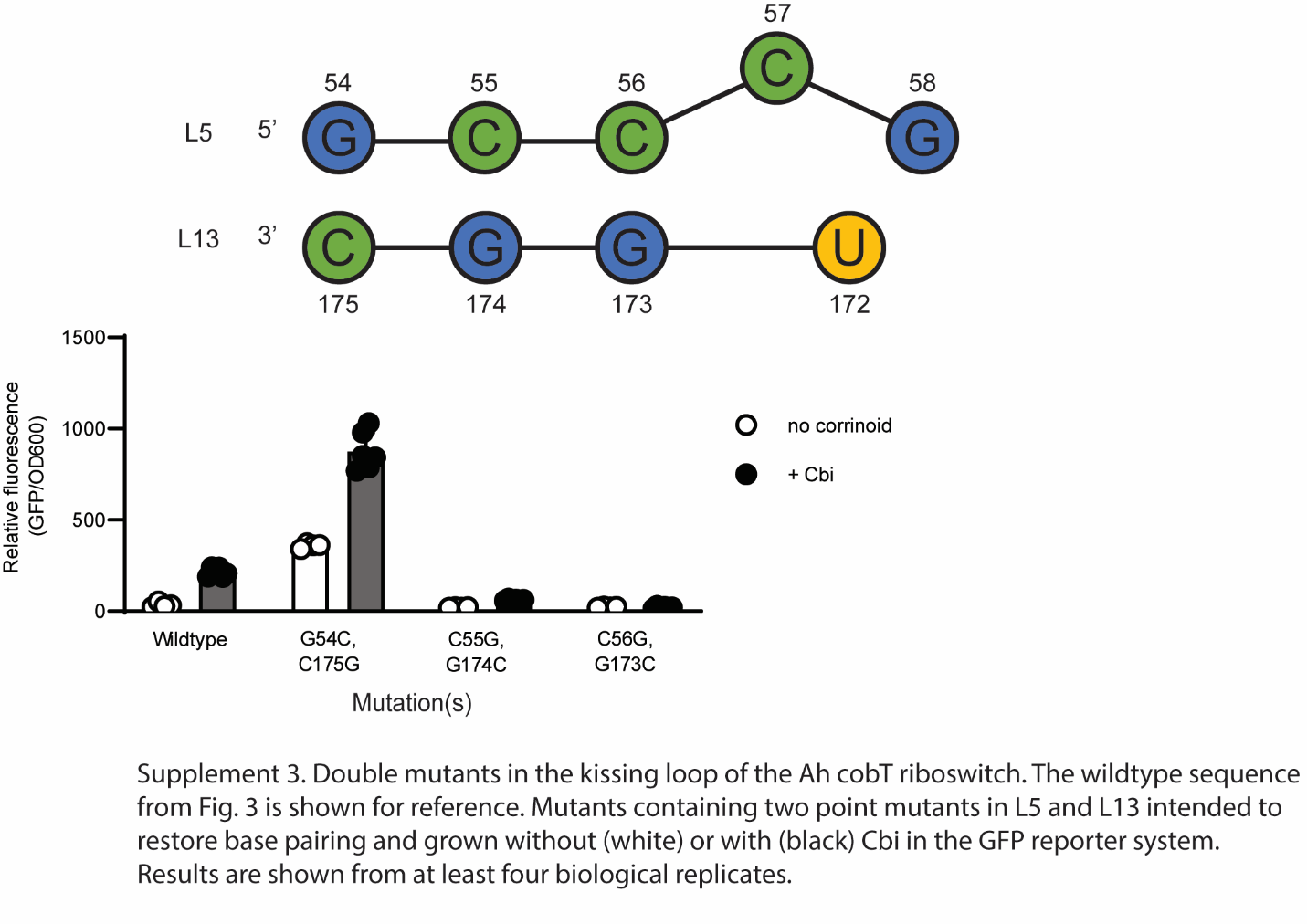
**

**Figure S3.** Double mutants in the kissing loop of the *A. halodurans cobT* riboswitch. The wildtype sequence from Figure 3 is shown for reference. Mutants containing two point mutants in L5 and L13 intended to restore base pairing were assayed in the *B. subtilis* GFP reporter system without (white) or with (black) addition of 100 nM Cbi. Data from four or more biological replicates are shown; bars and error bars represent mean and standard deviation, respectively.

**
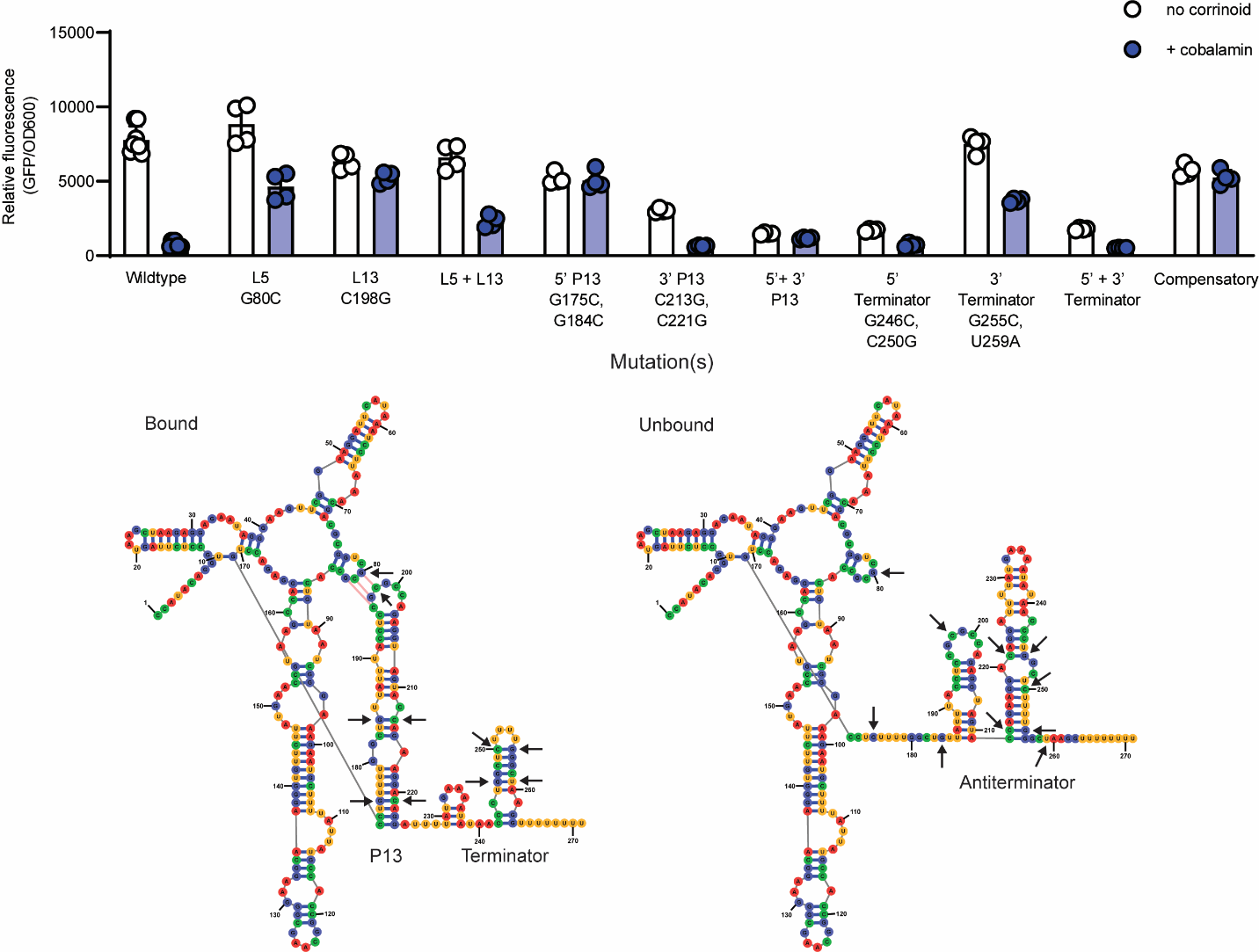
**

**Figure S4.** Dissection and predicted structure of the repressing *S. ovata cobT* riboswitch*.* The influence of point mutations in L5, L13, P13, and the terminator on gene expression was measured in the *B. subtilis* GFP reporter system without (white) or with (blue) addition of 100nM cobalamin. The label for each mutant includes the mutated region or the specific mutation, or both. Base numbering is relative to the first base in the structure. Models constructed manually and guided by computer-generated predictions using the StructureEditor program are shown below (22). Bases are color coded (adenine, red; guanine, blue; uracil, yellow; cytosine, green). Black arrows show the locations of the mutations made in this study. Data from four or more biological replicates are shown; bars and error bars represent mean and standard deviation, respectively.

**
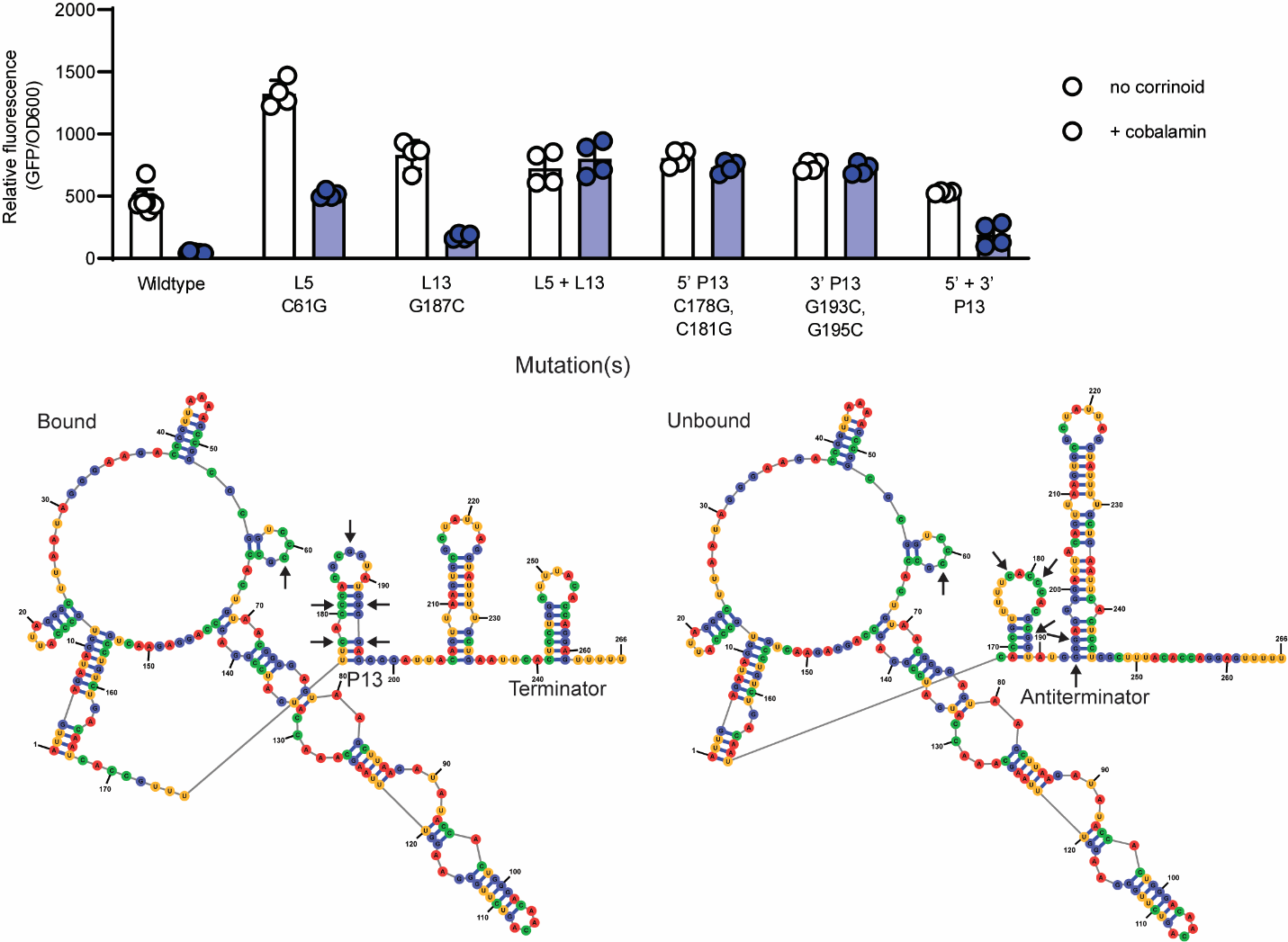
**

**Figure S5.** Dissection and predicted structure of the repressing *S. ovata nikA* riboswitch. The NikA protein has been annotated as a nickel transporter in *E. coli* but its function in *S. ovata* has not been reported. Mutations in the predicted kissing loop and P13 of the *S. ovata nikA* riboswitch grown without (white) or with (blue) addition of 100 nM cobalamin. The label for each mutant includes the mutated region or the specific mutation, or both. Base numbering is relative to the first base in P1. Models constructed manually and guided by computer-generated predictions using the StructureEditor program are shown below (22). Bases are color coded (adenine, red; guanine, blue; uracil, yellow; cytosine, green). Black arrows show the locations of the mutations made in this study. Data from four or more biological replicates are shown; bars and error bars represent mean and standard deviation, respectively.

**
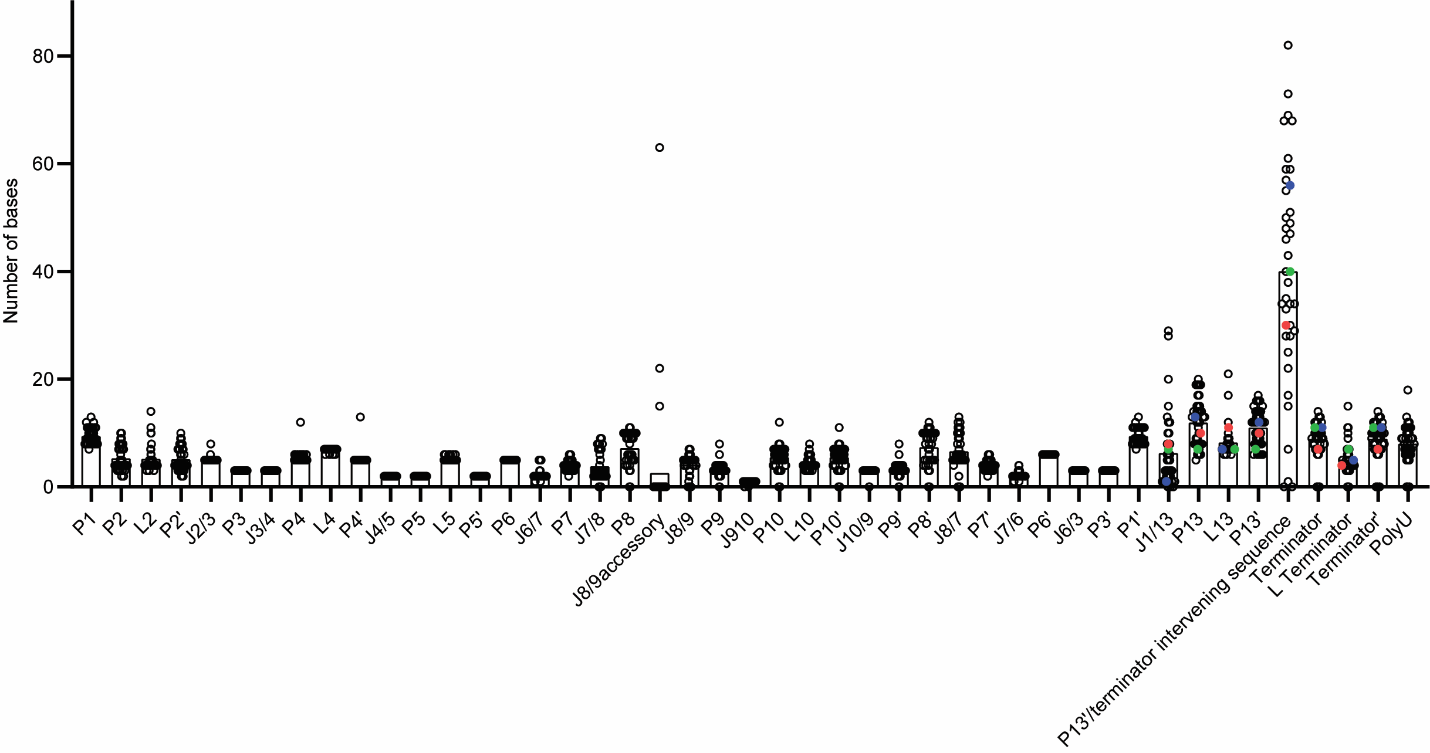
**

**Figure S6.** Diversity in the subdomain lengths in corrinoid riboswitches. Each point represents the length of the indicated region in one of 38 corrinoid riboswitches based on the multiple sequence alignment reported in Kennedy et al. 2022. Paired stems (P), loops (L), and junctions (J) are labeled. Colored dots in the expression platform subdomains show the location of the repressing *P. megaterium metE* (blue), *S. ovata cobT* (green), and *S. ovata nikA* (red) riboswitches. Bars represent the mean length. The ' label represents the 3’ side of a stem; the 5’ side is unlabeled.

**
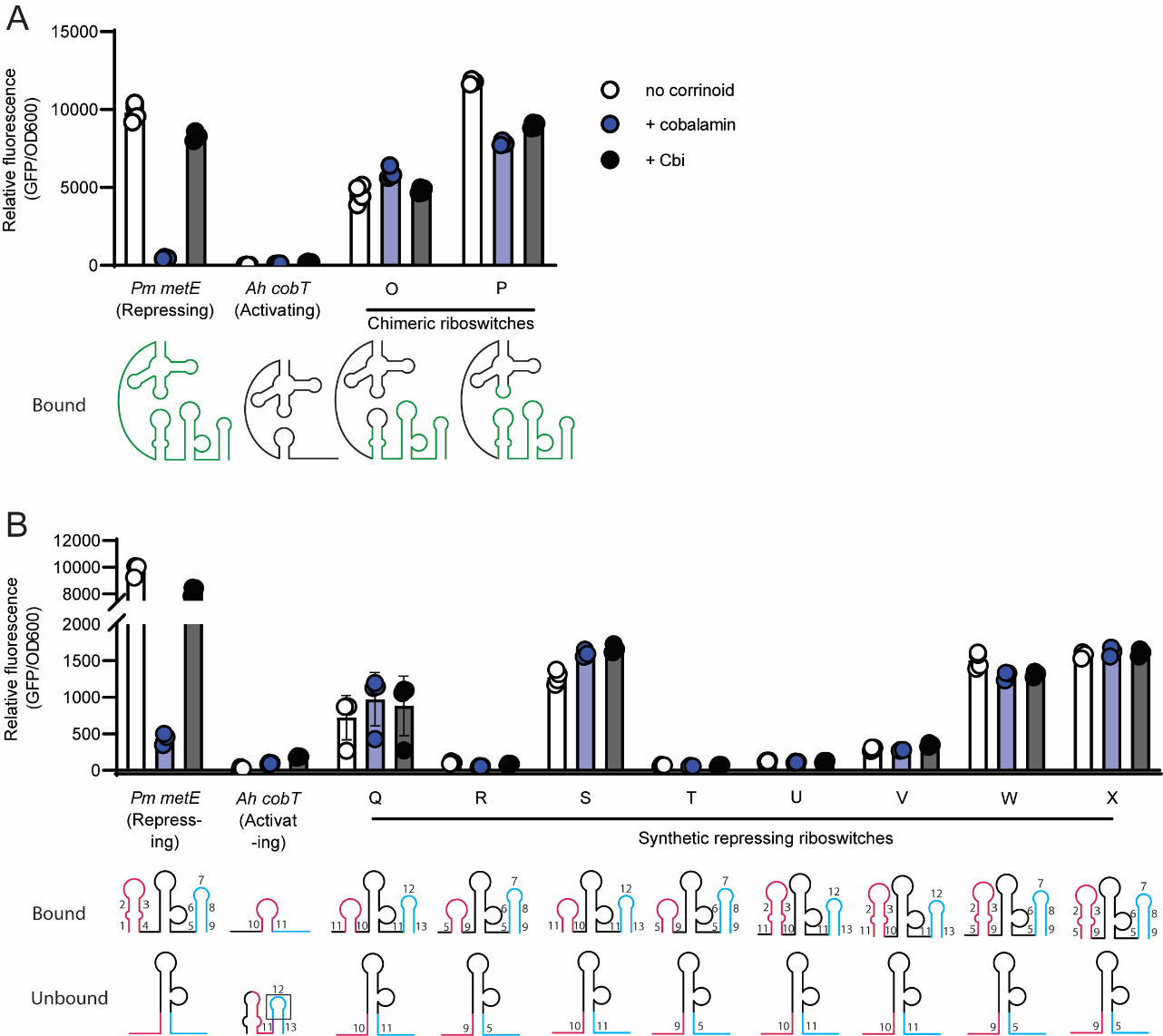
**

**Figure S7.** Chimeric and synthetic repressing riboswitches composed of the repressing *P. megaterium metE* and activating *A. halodurans cobT* riboswitches. A) Chimeric riboswitches were constructed by fusing the *A. halodurans cobT* aptamer with the *P. megaterium metE* expression platform, and gene expression was measured in the *B. subtilis* GFP reporter system with no corrinoid (white), or with addition of 100 nM cobalamin (blue), or Cbi (black). *P. megaterium metE* riboswitch sequences are shown in green and *A. halodurans cobT* sequences in black in the diagrams below, depicting the effector-bound conformation. Kissing loops were preserved by changing either L5 (Riboswitch P) or L13 (Riboswitch O). (B) Synthetic riboswitches were constructed by fusing parts of P13 and the terminators of the *P. megaterium metE* and *A. halodurans cobT* riboswitches. The diagrams below depict the expression platform of each riboswitch construct in the bound (top) and unbound (bottom) conformations. Numbers represent sequences from P13 (pink) and the terminator (blue). Data from four or more biological replicates are shown; bars and error bars represent mean and standard deviation, respectively.
